# From Exposure to Dysfunction: The Intestinal Toxicity of Per- and Polyfluoroalkyl Substances

**DOI:** 10.3390/toxics14010039

**Published:** 2025-12-29

**Authors:** Kashi Brunetti, Giulia Serena Galletti, Elisabetta Catalani, Davide Cervia, Simona Del Quondam

**Affiliations:** 1 ASST Fatebenefratelli Sacco Hospital, 20157 Milano, Italy; kashi.brunetti@unitus.it; 2 Department for Innovation in Biological, Agro-Food and Forest Systems (DIBAF), University of Tuscia, 01100 Viterbo, Italy; giuliagalletti06@gmail.com; 3 Department of Life Science, Health, and Health Professions, Link Campus University, 00165 Roma, Italy; e.catalani@unilink.it

**Keywords:** PFAS, gastrointestinal damage, intestinal barrier dysfunction, gut microbiota, oxidative stress, inflammation, inflammatory bowel disease

## Abstract

Per- and Polyfluoroalkyl substances (PFAS) are highly persistent synthetic chemicals increasingly associated with adverse health outcomes. The gastrointestinal tract represents both a major route of exposure and a key target of PFAS toxicity. This review integrates updated evidence on how PFAS compromise intestinal homeostasis through interrelated structural, metabolic, and immunological mechanisms. PFAS disrupt epithelial integrity by down-regulating tight-junction proteins, inducing oxidative stress, and activating inflammasome signaling. Concurrently, metabolic reprogramming and PFAS-driven microbial dysbiosis contribute to barrier dysfunction and altered production of signal/metabolic molecules. These alterations may link environmental exposure to chronic intestinal inflammation and increase susceptibility to inflammatory bowel disease and related metabolic disorders. By synthesizing recent findings, key mechanistic gaps were highlighted also emphasizing the need for integrative experimental and translational studies to refine risk assessment in humans and develop preventive and therapeutic strategies.

## 1. Introduction

Per- and Polyfluoroalkyl substances (PFAS) are a broad class of man-made fluorinated compounds whose carbon–fluorine backbone makes them highly resistant to thermal, chemical and biological degradation. Due to this inherent stability, they persist in environmental and biological systems for years, earning them the nickname “forever chemicals”. This remarkable stability, combined with a tendency to bioaccumulate and to spread across multiple environmental compartments, makes PFAS one of the most widespread and challenging classes of contaminants to remove. Understanding how these compounds interact with biological systems, particularly with the intestinal barrier, is therefore essential to assess their impact on human and environmental health.

Of the many PFAS currently recognized, Perfluorooctane sulfonate (PFOS) and Perfluorooctanoic acid (PFOA) remain the best characterized because of their widespread industrial use in non-stick, waterproof, and heat-resistant products [1]. Perfluorohexanoic acid (GenX) was introduced as a short-chain replacement for legacy PFAS to reduce their environmental and biological persistence. However, recent studies have demonstrated that, despite its shorter half-life, GenX exhibits toxicokinetic features comparable to its predecessors, including bioaccumulative potential and significant hepatic and intestinal toxicity in murine models [2,3].

Human exposure to PFAS occurs primarily via oral routes, through ingestion of contaminated drinking water, food, and household dust [4]. Consequently, the intestine functions not only as a primary site of absorption but also as a direct target of PFAS toxicity. Several studies have reported alterations in intestinal physiology following PFAS exposure, including epithelial barrier dysfunction, immune imbalance, and changes in microbial composition [5,6]. The intestinal barrier is a dynamic and highly specialized structure that maintains a delicate equilibrium between two complementary functions: the selective absorption of essential nutrients and the defense against pathogens and environmental toxins. When this integrity is disrupted, increased intestinal permeability, chronic inflammation, and ultimately inflammatory bowel diseases (IBD) may occur [7,8]. Evidence indicates that PFAS impair the structural integrity of the intestinal epithelium by down-regulating junctional proteins such as claudins and occludin, thereby increasing paracellular permeability [5]. These changes are frequently accompanied by a localized inflammatory response, marked by the upregulation of pro-inflammatory cytokines, and by intestinal dysbiosis that perpetuates barrier dysfunction [9].

Following a previous comprehensive review [4], this paper aims to provide an updated and focused picture of the cellular and molecular mechanisms through which PFAS compromise the intestinal barrier, highlighting the associated immunological, metabolic, and clinical implications across different experimental models. A focused literature search was conducted using a web-based approach (Scopus, PubMed, Google Scholar) to identify peer-reviewed studies more relevant to the intestinal toxicity of PFAS. The search included multiple keyword combinations such as “PFAS”, “intestinal damage”, “intestinal barrier”, “gut microbiota”, “intestinal metabolism”, “oxidative stress”, “gut epithelium”, intestinal inflammation/immunology”, “therapeutical approach”, and related terms. The abstracts of the articles were screened to determine whether they should be included in or excluded from the review. To be included, the article must have discussed original data and been published in a peer-reviewed journal. Articles published in English between 2018 and late 2025 were primarily considered, while earlier studies were included when necessary to provide mechanistic or historical context. Reference lists of selected papers were also screened to capture additional relevant sources. The search was conducted to generate an integrating, narrative synthesis of the current scientific knowledge on PFAS-induced intestinal alterations rather than systematic evidence mapping.

## 2. PFAS: Relevant Chemical and Physical Characteristics

PFAS constitute a vast family of organofluorine compounds widely used in industrial and domestic settings for their unique resistance and repellent properties. They are characterized by alkyl chains in which hydrogen atoms are wholly or partially replaced by fluorine atoms. This configuration confers them extraordinary chemical and thermal stability, related to the strength of the carbon–fluorine bond, one of the most robust in organic chemistry [4]. The presence of a hydrophobic perfluorinated tail and a hydrophilic terminal group, such as a carboxylate or sulfonate, confers on these molecules an amphiphilic behavior, similar to that of surfactants. This allows PFAS to interact readily with cellular membranes and plasma proteins, particularly albumin, promoting their distribution within the organism [9]. These same properties explain their persistence in ecosystems: low volatility, high surface tension and resistance to chemical and biological degradation enable PFAS to accumulate in soils and aquatic environments, propagate along the food chain and make them among the most difficult environmental contaminants to remove [4]. Numerous studies have documented the ability of PFAS to accumulate along aquatic food webs, including invertebrates, fish, birds, and marine mammals [10]. This biomagnification process is of particular concern because it can lead to secondary exposure to apex predators, including humans [11].

From a physiological standpoint, their chemical characteristics give PFAS a remarkable ability to cross biological barriers, including the intestinal barrier. Within the epithelium, such compounds can directly interact with membrane phospholipids, modifying fluidity and the organization of lipid domains [6,12]. This interaction, together with their surfactant nature, can alter the viscosity and composition of the mucous layer, reducing the protective efficacy of mucin and favoring an increase in epithelial permeability.

Carbon chain length represents a determining factor in defining the chemical and toxicological behavior of PFAS. Long-chain compounds, such as PFOS and PFOA, show greater affinity for lipid tissues and a more stable binding to plasma proteins, promoting bioaccumulation and a biological half-life that, in humans, can extend from several months to many years. Conversely, short-chain PFAS such as Perfluorobutanoic acid (PFBA), Perfluorobutane sulfonic acid (PFBS) or GenX, are considered more “eco-compatible” and water-soluble. These PFAS alternatives are associated with faster absorption, less distribution into tissues, and greater elimination versus long-chain PFAS, resulting in lower systemic exposure [4]. However, their greater mobility in biological compartments facilitates systemic distribution and interaction with different cellular targets, suggesting that they are not necessarily less toxic than their predecessors [13,14]. Although limited studies are available on their biological impact, such “next-generation” substitutes do not represent a risk-free solution, also because their increased solubility may favor diffusion into aquatic compartments and soft tissues. To make reading easier, from now on, when we refer to short-chain PFAS in the text, this will be specified.

Taken together, the physicochemical characteristics of PFAS, including remarkable molecular stability, amphiphilic nature, ability to bind plasma proteins and interaction with membranes and receptors, explain both their environmental persistence and their bioactivity in tissues. These properties make PFAS xenobiotics of toxicological relevance, capable of interfering with intestinal physiology even at relatively low exposure concentrations.

## 3. Human Exposure to PFAS

Because of their exceptional stability and extensive industrial applications, PFAS have emerged as some of the most pervasive and enduring contaminants in both environmental and human systems. Numerous studies have demonstrated widespread human exposure to PFAS across populations worldwide [15,16,17]. In the general population, ingestion represents the main exposure pathway, positioning the gastrointestinal tract as both the entry point and one of the first organs affected by PFAS toxicity. The most common sources include drinking water, contaminated food, household dust, and a wide range of consumer products such as food packaging, water-repellent textiles, and non-stick coatings containing fluorinated compounds [18,19].

Among the various exposure pathways, water contamination represents one of the most documented and globally relevant concerns. Industrial activities and consumer applications, such as the production of firefighting foams, coatings, and textiles, discharge PFAS into the environment, where they readily percolate into surface and groundwater systems. Investigations conducted near industrial sites or landfills containing fluorinated waste have reported significant concentrations of PFOS and PFOA in public water supplies, raising increasing public health concerns and driving the definition of progressively stricter safety limits [20]. In this contest, long-term ingestion of contaminated water has been linked to elevated serum PFAS levels in the general population, even at concentrations lower than current guideline limits.

Dietary exposure is another major pathway. In animals, PFAS tend to bioaccumulate in fish and other aquatic species from contaminated habitats and have also been detected in livestock products from exposed regions. Additionally, contact with food packaging treated with fluorinated coating can further contribute to contamination. Their ability to bind proteins and lipids facilitates transfer along the food chain, resulting in progressive accumulation in animal-derived products and, consequently, in humans [21,22]. In this context, it has been recently estimated that dietary intake accounts for more than 70% of total PFAS exposure in the European population, with fish as the principal contributor [23].

A frequently overlooked source of exposure derives from indoor dust. Indoor environments such as homes and offices can serve as reservoirs for PFAS emitted from treated fabrics, carpets, paints, and electronic materials. These substances are slowly emitted into the air or onto particulate matter and can be incidentally ingested, posing particular concern for children, who display more frequent hand-to-mouth behavior and increased physiological vulnerability. Studies conducted in European households have shown a direct correlation between PFAS levels in dust and serum concentrations in residents, indicating that non-dietary exposure can meaningfully contribute to total body burden [24]. This exposure pathway is especially relevant indoors, where the low volatility of PFAS favors their deposition on surfaces and absorbent materials.

### 3.1. Human Bioaccumulation Insights

After ingestion, PFAS are efficiently absorbed in the small intestine via passive diffusion and specific anion transporters. Once in circulation, they exhibit strong affinity for plasma proteins which drive their accumulation in highly perfused organs [25,26]. Analysis of PFAS presence in human blood and major target organs showed that plasma levels reflect systemic exposure and that the liver acts as an important accumulation site for these compounds [27]. For instance, concentrations of PFOA and PFOS were measured in different organs collected post-mortem from seven non-occupationally exposed individuals in Northern Italy [28]. Results showed higher accumulation in the liver (3.1–13.6 ng/g), kidneys (3.5–6.4 ng/g), and lungs (3.8–7.9 ng/g), followed by pituitary gland (2.0–7.6 ng/g), thyroid (2.3–3.1 ng/g), pancreas (1.3–3.5 ng/g), and gonads (1.9–3.4 ng/g). Lower concentrations were detected in adipose tissue (1.4–1.7 ng/g), brain (0.5–1.3 ng/g), basal ganglia (0.3–1.2 ng/g), and skeletal muscle (0.6–1.0 ng/g). These data, based on the analysis of a single pooled sample for each compound, suggest a differential distribution of PFAS among human tissues. In this respect, intestinal microbial community may represent an additional factor contributing to the persistence and bioavailability of PFAS. Bacterial strains such as Bacteroides uniformis and Escherichia coli can accumulate PFAS at high concentrations, and in murine models colonized with typical human bacteria, bacterial bioaccumulation influenced the amount of PFAS excreted in feces [29].

Regulatory authorities such as the European Food Safety Authority (EFSA) and the USA Environmental Protection Agency (EPA) have consequently established very stringent limits, typically in the nanogram-per-liter range, to restrict human exposure. EFSA has established a safety threshold for intake as the sum of the four PFAS at 4.4 ng/kg body weight per week, corresponding to an internal blood level of 6.9 μg/L [30]. However, the remarkable environmental persistence and bioaccumulative potential of PFAS make it difficult to prevent long-term exposure, even under increasingly stringent regulations [21]. Indeed, in highly contaminated areas, serum PFOA levels may reach 83.0 ng/mL in residents [31] and exceed 5000 ng/mL in occupational settings [32].

The liver represents a primary target organ for the storage of long-chain PFAS, and experimental evidence of toxicity includes lipid infiltration in hepatocytes and specific induction of cytochrome P450 pathway. A major contribution to understanding PFAS distribution and toxicity concerns their effects on hepatic transporters. Recent evidence indicates that PFAS can interact with membrane proteins in the liver, modulating the influx and efflux of xenobiotics and thereby influencing hepatic accumulation and systemic persistence [33]. These interactions are also associated with metabolic changes, such as increased serum cholesterol levels in exposed adults, reinforcing the role of the liver as a primary target organ in PFAS bioaccumulation and toxicity processes.

PFAS display marked variability in toxicokinetic characteristics, distribution, and elimination across species and among individuals, influenced by factors such as age, sex, and metabolic status [34]. The interactions with proteins such as albumin and fatty acid–binding proteins [35,36] explain the prolonged biological half-life of PFAS, which for compounds such as PFOS and PFOA can extend to several years in humans while it is hours/days in rodents [37,38,39,40,41]. Binding to transthyretin, the transport protein of thyroid hormone, has also been demonstrated [42]. Recent experimental evidence obtained in a human induced pluripotent stem cell-derived intestinal epithelial cell model demonstrated that exposure to PFAS can impair epithelial functionality, reducing tight junction integrity and altering the expression of key genes involved in intestinal barrier maintenance [43]. These data suggest that the intestine is not merely a passive absorption site but also a primary target of direct toxicity.

### 3.2. Environmental Exposure and Animal Studies

Comparison between serum or tissue PFAS levels measured in animals and human biomonitoring data allows consideration of marked inter-species pharmacokinetic differences, particularly the greater persistence and substantially longer half-life of PFAS in humans compared with animal models [44,45]. In this context, the concentrations of PFAS employed in experimental models are often higher than those typically encountered in environmental human exposure; however, their biological relevance is generally interpreted based on internal burden rather than the external administered dose alone. For instance, mice exposed via drinking water to a mixture of five PFAS containing PFOA at a concentration of 2 mg/L for 84 days reached serum PFOA levels of 7.3 μg/mL [46], comparable to those reported in occupationally exposed human populations, where serum concentrations ranged from 1.13 to 12.9 μg/mL [32,47]. On the basis of this comparability in terms of internal body burden, a recent study on the mechanisms of PFAS-induced colon toxicity selected drinking water PFOA concentrations of 0, 0.2, and 2 mg/L for mice administration [48]. Also, toxicological studies published on GenX to assess the dose to be administered showed that, when the dose was less than 30 mg/kg, there were no effects on foetal birth weight after pregnant rats were exposed to GenX from the eighth day of gestation to the second postnatal day [49,50]. When the exposure dose was raised to 110 mg/kg, half of the foetuses died at birth. Based on these data, the highest exposure dose of 100 mg/kg was chosen to explore whether GenX produces adverse effects on offspring development in Sprague–Dawley rats and the underlying mechanisms [49]. Of interest, a range of PFOA doses between 1 and 40 mg/kg/day was recently administered with the aim of achieving stable and dose-dependent serum concentrations in mice, thereby enabling quantitative analysis of dose–response relationships for maternal and developmental endpoints. In this context, the relatively high doses employed in animal studies are not intended to directly replicate environmental human exposure, but rather to mimic internal body burdens that may be reached in humans following chronic, long-term exposure [51]. These data support the use of more quantitative dose–response modeling approaches, such as the Benchmark dose (BMD) method, which estimates a dose corresponding to a predefined change in effect and derives a confidence-bounded point of departure. In contrast to the NOAEL (No Observed Adverse Effect Level), that defines the highest tested dose of a substance at which no significant harmful health effects are observed in the exposed population, the BMD approach uses the entire dose–response dataset, allows interpolation between tested doses, and provides an explicit characterization of statistical uncertainty and effect magnitude, making it generally more informative for risk assessment and better suited to bridge experimental exposures with real-world human scenarios [52].

### 3.3. PFAS Combinations as a Critical Aspect of Environmental Toxicology

In real-world scenarios, humans are rarely exposed to a single PFAS. Mixed exposures from various sources can interact additively or synergistically, modifying the bioavailability and toxicokinetics of individual compounds [53]. Human biomonitoring studies have consistently detected multiple PFAS in blood, urine, and breast milk across populations, underscoring the pervasive and persistent nature of complex and mixed exposures [54]. However, interaction mechanisms of the “cocktail effect” remain largely unclear, making it challenging to establish reliable health-protective threshold values. The combined exposure to PFAS has been shown to produce toxic responses in vitro systems that cannot be predicted on the basis of individual compounds, suggesting a convergence of mixtures on common toxicity pathways relevant to intestinal barrier integrity and mucosal inflammation regulation [55]. In addition, a recent study demonstrates that mixtures of PFAS, tested at environmentally relevant concentrations comparable to those found in human blood, induced significant neurotoxic effects even in the absence of marked effects of individual compounds, highlighting predominantly additive and, in some cases, potentially synergistic interactions [56]. These effects were associated with the alteration of fundamental cellular processes, such as calcium homeostasis, mitochondrial function and intracellular signaling pathways, mechanisms that are highly conserved even in the intestinal epithelium. These data indicate that PFAS-induced intestinal damage may be amplified by exposure to mixtures, contributing to cumulative alterations in barrier function, immune response, and tissue homeostasis, and highlight how toxicological approaches based on single substances may underestimate the actual risk associated with PFAS.

In summary, PFAS exposure is widespread, continuous, mixed, and driven by multiple sources, with the oral route representing the principal point of entry into the body. The high efficiency of intestinal absorption, combined with the long biological half-life of these compounds, enhances their persistence and toxic potential. PFAS, due to their amphiphilic structure (a hydrophobic fluorocarbon chain and a hydrophilic functional group), readily bind to lipoproteins and bile acids or form micelles, remaining suspended in gastrointestinal fluids and thereby enhancing bioaccessibility. Moreover, PFAS carbon chain length may influence their behavior during digestion [57]. In addition to documented systemic toxicity, PFAS exposure, particularly to PFOS, has been associated with intestinal tissue inflammation and, in some preclinical models, with signals potentially related to gastrointestinal carcinogenic processes. These findings suggest that the intestine is not merely an absorption site but can be directly affected by PFAS, with potential implications for chronic inflammation and related diseases [58]. In this respect, the gut microbiota itself can accumulate PFAS, influencing their biodistribution and potentially their intestinal clearance or metabolism. Conversely, chronic PFAS exposure, particularly to PFOS, affects gut microbiota composition and function, thus altering immune responses and systemic inflammation [58]. Taken together, these characteristics justify the growing focus on PFAS impacts on intestinal barrier integrity, microbiota composition and function, and local immune mechanisms, aspects that will be explored in detail in the following sections.

## 4. Effects of PFAS on the Intestinal Barrier

### 4.1. Structural Damage and Molecular Alterations

Maintenance of intestinal barrier function depends on the coordinated arrangement of epithelial junctions that control paracellular transport and sustain the segregation between luminal contents and systemic compartments. Chronic exposure to PFAS, particularly PFOS and PFOA, can impair this architecture through morphological alterations, transcriptional modulation, and cellular dysfunctions that broadly influence intestinal epithelial physiology.

Experimental evidence indicates that PFAS directly interferes with the expression of key junctional components, including occludin, claudins, and the tight junction protein 1 (Tjp1). In murine models, prenatal or postnatal exposure to low doses of PFOS (0.36 mg/kg) has been associated with a marked reduction in both gene and protein expression of Tjp1 and claudin-4, together with increased circulating zonulin levels, a recognized biomarker of enhanced intestinal permeability [5]. These findings suggest that early-life or sub-toxic exposures may durably disrupt epithelial maturation and function, predisposing to inflammatory conditions or dysbiosis. Studies conducted in adult mice exposed to short-term (10 days) oral administration of PFOA demonstrated a dose-dependent modulation of genes involved in tight junction assembly, including several claudin isoforms (*Cldn2*, *Cldn3*, *Cldn4*, *Cldn8*, *Cldn12*, and *Cldn15*), *Tjp1*, *Tjp2*, and occludin [6]. This study also reported alterations in the intestinal expression of DNA methylation–related genes (*Dnmt1*, *Dnmt3a*, *Dnmt3b*, *Tet1-3*), indicating a direct impact on the epigenetic machinery sustaining intestinal homeostasis.

Human-derived intestinal epithelial lines, notably Caco-2 and HT-29, recapitulate key physiological features of the intestinal mucosa, such as the establishment of tight junctions, cell polarization, and absorptive capacity, making them valuable systems for mechanistic studies of PFAS toxicity [59]. Exposure of these cell systems to PFOS or PFOA consistently decreases transepithelial electrical resistance and downregulates major junctional proteins, including occludin, claudin-1, and Tjp1, reflecting a direct compromise of epithelial cohesion [60]. Cell death also represents a contributing mechanism to barrier dysfunction [61].

Since epigenetic abnormalities have been linked to the onset of human IBD in experimental models [62], PFAS-induced modifications of DNA methylation patterns may play a relevant pathogenic role. Notably, such alterations appeared particularly evident in the small intestine, where high absorptive capacity favors PFAS retention and tissue sensitivity. At the molecular level, PFAS-induced effects appear to reflect transcriptional regulation driven by oxidative stress and nuclear factor kappa-light-chain-enhancer of activated B cells (NF-κB)-dependent inflammatory pathways, contributing to the loss of epithelial barrier integrity [62]. Increased phosphorylation of the NF-κB p65 subunit and degradation of the kinase IκBα observed in Caco-2 cells confirm that inflammatory activation seen in vivo systems is a major mechanism driving epithelial toxicity [63].

Chronic PFOA exposure exerts even broader effects when sustained over time. In a murine model receiving continuous administration at concentrations of 0, 0.2, 2 mg/L for 180 days via drinking water, colon mucosa exhibited features of persistent inflammation, including crypt disorganization and alterations in the population of intestinal stem cells [48]. Transcriptomic profiling revealed suppression of the Wnt pathway, essential for epithelial renewal, together with activation of pro-inflammatory signaling cascades such as Toll-like receptor-2 (TLR2), TLR3, and the NOD-, LRR- and pyrin domain-containing protein 3 (NLRP3) inflammasome. Moreover, PFOA triggered PPAR-dependent signaling, leading to abnormal lipid metabolism. Collectively, these findings suggest that prolonged PFAS exposure may compromise the regenerative capacity of the colonic epithelium and promote chronic inflammatory processes.

Chronic exposure to PFOA in the fruit fly Drosophila melanogaster results in delayed larval development, reduced adult body size, and altered energy metabolism. These effects appear to be closely linked to intestinal inflammation. Increased levels of Eiger and Unpaired3 in the intestinal epithelium, as well as their modulation via genetic knockdown, confirm the crucial role of these inflammatory factors. Overall, the data outline a functional axis of PFOA–intestinal inflammation–sleep alterations, with the intestinal epithelium emerging as the primary target and intestinal inflammation as the central mediator of the observed neurotoxicity [64]. This model therefore represents a simplified yet powerful system for genetic screening and exploring the evolutionary conservation of toxicological mechanisms.

Additionally, morphological studies in adult rats exposed to PFOS for 15 days demonstrated significant structural lesions even at relatively low doses (1 mg/kg), including reduced villus height and crypt depth, increased epithelial apoptosis, macrophage and neutrophil accumulation, and infiltration of pro-inflammatory cytokines [65]. These observations indicate that substantial histopathological alterations may occur at exposure levels comparable to those found in the environment. Beyond overt structural injury, PFAS can induce more subtle yet functionally relevant disruptions, such as mitochondrial dysfunction and loss of membrane potential. These processes accelerate enterocyte apoptosis and impair cortical actin remodeling, critical determinants of epithelial cohesion. In PFOS-exposed murine models, such phenomena have been associated with elevated reactive oxygen species (ROS) production and transcriptional changes in genes regulating lipid metabolism and epithelial turnover [66].

Taken together, current evidence delineates a coherent mechanistic framework: PFAS weakens the intestinal barrier through combined structural injury and transcriptional dysregulation, with effects detectable even at low exposure levels and intensified by chronic intake. The resulting barrier compromise represents a potential initiating event for inflammatory and tissue-remodeling processes, considered central mechanisms in the development of PFAS-associated chronic intestinal disorders.

### 4.2. Activation of the Inflammatory Response

Disruption of the epithelial barrier by PFAS elicits a multifaceted inflammatory reaction engaging both innate and adaptive immune pathways. The activation of these immune pathways suggests that inflammation represents a central pathogenic mechanism through which PFAS exert their systemic toxicity. In this context, the intestine emerges not only as a direct target organ, but also as a potential initiator of immune–inflammatory responses at the systemic level. In addition to intestinal effects, some studies have reported systemic alterations, such as pancreatic tissue damage and impaired carbohydrate metabolism, induced by both traditional PFAS and their new-generation substitutes [67,68].

Experimental evidence indicates that exposure to PFOS and PFOA promotes a strong pro-inflammatory state characterized by increased levels of key inflammatory cytokines, including Tumor necrosis factor-α (TNF-α) and Interleukin-6 (IL-6), which are pivotal mediators of intestinal inflammation. In rats exposed to PFOS for 15 days at 10 mg/kg and 1 mg/kg, a marked increase in macrophage and neutrophil infiltration, elevated expression of TNF-α and IL-6, and enhanced epithelial apoptosis in the jejunum were observed [65]. Additional findings from aquatic models have corroborated the ability of PFAS to disrupt intestinal barrier function. In Zebrafish with trinitrobenzenesulfonic acid-induced colitis, PFOS administration amplified the expression of pro-inflammatory cytokines TNF-α and IL-1β and promoted neutrophil recruitment into the intestinal tissue of larvae. This immune cell infiltration increased intestinal permeability and also induced an extra-intestinal T-cell response to PFOS during colitis, indicating that PFOS exacerbates inflammation-induced tissue damage and suggesting a synergistic interaction between direct PFAS toxicity and local immune activation [69]. These findings indicate that PFAS not only act as primary pro-inflammatory stimuli but can also potentiate pre-existing inflammatory responses, potentially increasing susceptibility to chronic inflammatory conditions such as colitis and favoring preneoplastic transformations in a context of persistent inflammation.

In parallel with canonical cytokine production, PFAS activate critical components of innate immunity through stimulation of cytosolic inflammasomes. PFOS exposure triggers NLRP3-inflammasome activation, leading to caspase-1 cleavage and the maturation of the pro-inflammatory cytokines IL-1β and IL-18 [48,70]. Also, PFOS impairs mitochondrial function, triggering the selective release of mitochondrial DNA into the cytosol without concurrent nuclear DNA release, a key event for AIM2 (Absent in melanoma 2) inflammasome activation [32]. AIM2 is a member of innate immune sensors that detects altered or mislocalized DNA molecules within the cytosolic compartment, arguably the most conserved molecules in living organisms. Under conditions of pre-existing oxidative stress, inflammasome activation is further enhanced, generating a positive feedback loop between ROS production and inflammatory mediator release [71]. Recent evidence indicates that PFOS can activate AIM2 independently of NLRP3, pointing to parallel engagement of multiple cytosolic inflammatory platforms. This phenomenon underscores the ability of PFAS to disrupt evolutionarily conserved defense mechanisms, resulting in chronic hyperactivation of innate immunity and contributing to tissue injury. NF-κB signaling acts as a pivotal regulatory hub within the inflammatory cascade initiated by PFAS exposure. Studies in human intestinal epithelial cell lines have shown that PFOS exposure induces phosphorylation of the p65 subunit and degradation of IκBα, events promoting nuclear translocation of NF-κB and expression of pro-inflammatory genes such as *TNFA* and *IL6* [70]. This process is closely associated with increased intracellular Ca^2+^ and Protein kinase C (PKC) activation, highlighting the complexity of intracellular signaling networks. Of interest, chronic NF-κB activation may compromise intestinal homeostasis and promote pro-oncogenic phenotypes by enhancing cell proliferation and inhibiting early apoptosis [72]. Recent findings demonstrated that prenatal exposure to PFOA or GenX induced hepatic injury and intestinal dysregulation in dams and exerted vertical effects on liver and gut health in offspring via activation of the TLR4/NF-κB/NLRP3 pathway, suggesting intergenerational consequences [2]. Overall, these studies highlight the pivotal role of Ca^2+^-PKC, NF-κB, NLRP3, and AIM2 in PFAS-induced toxicity and support their relevance as a potential therapeutic target.

The immune activation triggered by PFAS also involves recruitment of inflammatory cells into the intestinal mucosa. In multiple experimental models exposed to PFOS, increased macrophage and neutrophil infiltration was accompanied by enhanced ROS production and oxidative membrane damage [65,69]. In this respect, oxidative stress acts as a potent amplifier of inflammation by reinforcing inflammasome activation, creating a self-sustaining cycle in which ROS and pro-inflammatory cytokines intensify each other’s effects [71]. This mechanism contributes to the persistence of chronic intestinal inflammation, potentially predisposing to diseases such as ulcerative colitis. It has been reported that even chronic low-dose PFAS exposure in humans may subtly remodel immune balance by potentiating inflammatory pathways of both innate and adaptive immunity [73]. Increased activation of Natural Killer cells and expansion of memory T-helper subsets, together with a reduction in cytotoxic CXCR3^+^ effector-memory T cells, illustrate systemic immune dysregulation that may heighten susceptibility to inflammatory and autoimmune disorders in exposed populations.

The intestine is a critical sentinel organ whose integrity is essential for maintaining systemic homeostasis and preventing propagation of inflammatory signaling. Damage to the intestinal epithelium may be a key event in the systemic toxicity associated with PFAS exposure. Compromised epithelial integrity permits translocation of microbial products such as lipopolysaccharide (LPS) into circulation, thereby activating TLR4-dependent inflammatory signaling in distal tissues [74]. In models exposed to PFOA, significant increases in circulating LPS and pronounced NF-κB activation in both liver and brain have been documented, establishing a mechanistic connection between intestinal inflammation and systemic immune responses [69,75]. This state of subclinical endotoxemia may constitute an initiating mechanism underlying PFAS-associated metabolic dysfunction and neuroinflammation, providing biological plausibility to epidemiological associations with cognitive impairment and metabolic diseases.

### 4.3. Functional Alterations and Effects on Epithelial Metabolism

In addition to compromising the structural integrity of the barrier and promoting a persistent immune response, PFAS directly interfere with intestinal epithelial cell physiology. PFAS exposure interferes with essential epithelial processes, ranging from nutrient uptake and metabolic regulation to differentiation and regenerative capacity. Such dysfunctions not only exacerbate intestinal permeability and local homeostasis imbalance but can also contribute to the onset of systemic metabolic disorders. Collectively, these findings strengthen the concept that the gut represents a primary target and a critical hub in PFAS-driven toxicity, with consequences extending well beyond the gastrointestinal tract.

In particular, a well-characterized effect concerns the interference of PFAS with intestinal lipid metabolism. Studies in human intestinal organoids have shown that PFOS enhances fatty acid uptake through activation of peroxisome proliferator-activated receptor α (PPARα), a key regulator of β-oxidation and lipid transport. This activation is associated with increased expression of genes involved in lipid transport (*FABP1*, *CD36*), fatty acid oxidation (*ACOX1*, *PDK4*), and lipid droplet synthesis (*PLIN2*, *PLIN3*) [76]. In essence, PFOS reprograms epithelial metabolism toward enhanced lipid uptake and storage, a shift that may disturb intestinal and systemic energy balance.

Using PPARα knockout models has further clarified the role of this nuclear receptor in the toxic response to PFAS: the absence of PPARα attenuates the mitochondrial dysfunction and lipid absorption defects induced by PFOS, suggesting that it acts as a key mediator of the metabolic effects of these contaminants [77].

Notably, these molecular changes were not observed in response to PFOA, suggesting substantial mechanistic differences among PFAS. Variability in fluorinated chain length and nuclear receptor affinity may explain these discrepancies, reinforcing the need to assess the toxicological impact of each PFAS individually rather than as a single homogeneous chemical class. In another study human HepG2 hepatocytes exposed to PFAS with different structures (PFOA, PFOS, PFBA, and PFBS) displayed marked metabolic perturbations, mainly affecting lipid pathways, with greater toxicity observed for long-chain and sulfonate-containing compounds [78]. Gene expression analyses confirmed disruption of lipid regulatory pathways, accompanied by intracellular lipid accumulation, oxidative damage with subsequent lipid peroxidation, and reduced cell viability. Long-chain PFAS (PFOA and PFOS) showed higher toxicity than short-chain analogs (PFBA and PFBS), and sulfonates (PFOS, PFBS) were more potent than carboxylates of comparable length (PFOA, PFBA). Additionally, low-dose exposure to PFOA and PFOS (0.1–1 μM) in the nematode Caenorhabditis elegans triggered significant lipid accumulation, resulting in weight gain [79]. Lipid profiling revealed a decrease in the ratio of saturated to polyunsaturated fatty acids. Consistently, mutant screening and transcriptional analyses demonstrated significant regulation of lipid biogenesis-related genes including mdt-15, nhr-49, and fat-6 (involved in fatty acid desaturation), together with fasn-1 and dgat-2, key regulators of fatty acid and triglyceride synthesis.

PFAS effects extend beyond metabolism to include disruption of epithelial cell differentiation. Enteroendocrine cells play a crucial role in secreting gastrointestinal hormones such as glucagon-like peptide-1 and peptide YY, which regulate appetite, energy metabolism, and insulin sensitivity [80]. PFOS exposure in human organoid models resulted in a significant reduction in enteroendocrine cell abundance, assessed through chromogranin A expression [76]. This reduction suggests systemic consequences for glucose regulation and adiposity, potentially contributing to the epidemiological association between PFAS exposure, obesity, and type 2 diabetes.

PFAS also affect epigenetic regulation and intestinal stem cell function, which are essential for continuous mucosal renewal. Chronic exposure to PFOA for 180 days in rats inhibited the Wnt pathway, a key driver of epithelial stem cell proliferation and differentiation [48]. Transcriptomic analyses revealed alterations in genes involved in tight junction assembly and energy metabolism, indicating widespread impairment of tissue homeostasis. Histologically, such changes are manifested by disorganized crypts, shortened villi, and reduced mucosal regenerative capacity. These observations suggest that PFAS may modulate the epigenome of intestinal stem cells, reducing cellular plasticity and increasing vulnerability of the intestinal barrier to chronic injury.

Altered epithelial metabolism under PFAS exposure extends to mitochondrial dysfunction, impairing energy production and redox balance. In vitro studies on intestinal cells demonstrated that PFOS decreases mitochondrial respiratory capacity, promotes the accumulation of ROS, and induces loss of mitochondrial membrane potential [58]. The resulting mitochondrial deficits reduce ATP supply necessary for maintaining tight-junction assembly and mucus secretion, key determinants of barrier integrity. Furthermore, excess ROS can activate intrinsic apoptotic pathways, promoting epithelial cell loss and worsening tissue damage [81]. The impact of PFOS on mitochondrial activity was also investigated in placental trophoblasts, where these compounds are highly concentrated. PFOS exposure reduced basal and maximal respiration and ATP-linked oxidative phosphorylation by inhibiting complexes I, II, and III of the electron transport chain [82]. A decrease in mitochondrial number was also observed. At the transcriptional level, PFOS downregulated key regulators of mitochondrial biogenesis (Peroxisome-proliferator-activated receptor gamma coactivator 1-α, Nuclear respiratory factor 1 and 2) and impaired mitochondrial dynamics by suppressing genes involved in both fission and fusion processes.

In summary, converging evidence demonstrates that PFAS profoundly alter intestinal physiology. They not only damage epithelial structure but also reprogram metabolic and differentiation pathways, affecting nutrient absorption, tissue regeneration, and hormonal regulation. These alterations provide a plausible mechanistic framework linking chronic PFAS exposure to systemic metabolic disorders, including metabolic syndrome, obesity, and diabetes. Overall, the intestinal epithelium emerges as a central mediator of PFAS systemic toxicity, acting as both a primary target organ and a regulator of whole-body energy homeostasis.

## 5. Effects of PFAS on the Gut Microbiota

The gut microbiota forms a crucial ecological network that sustains mucosal balance, regulating immune response, and contributes to the host’s metabolic and energetic homeostasis. Alterations in its structure or function, collectively termed dysbiosis, have been linked to a wide spectrum of intestinal and systemic diseases. In recent years, a growing body of evidence has identified PFAS as a novel class of environmental contaminants capable of disrupting this delicate balance. Exposure to these compounds can lead to structural and functional changes in the microbial community, altering its metabolic and immunomodulatory capacity and potentially amplifying host physiological responses across multiple organ systems.

### 5.1. Compositional Alterations of the Gut Microbiota

A substantial amount of experimental evidence indicates that PFAS exposure can profoundly alter gut microbiota composition. In mice chronically exposed to PFOS, the Firmicutes/Bacteroides ratio declined, a shift typically correlated with inflammation and metabolic imbalance [66]. A reduction in this ratio has been linked to the onset of IBD, since Bacteroides exhibit pro-inflammatory properties through cytokine modulation, whereas Firmicutes generally exert anti-inflammatory effects that counteract IBD progression [83]. Using an in vitro human colonic model, exposure to a PFAS mixture modified community structure, with changes in the representation of bacterial genera such as Morganella and Bilophila [84]. Moreover, PFOS exposure has been associated with an increase in genera such as Clostridium and Streptococcus, while depleting Flavonifractor, Alistipes, and an unclassified Bacteroides genus [66]. Short-chain PFAS exposure in mice led to reduced abundance of beneficial taxa such as Lactobacillus, Enterococcus and Akkermansia, both essential for mucin synthesis and immunoregulatory signaling [9,85]. The reported increase in Bacteroidota, including genera such as Escherichia and Desulfovibrio, is particularly relevant because these microorganisms produce LPS, strong activators of innate immune responses. This microbial profile promotes inflammation and can magnify the direct harmful effects of PFAS on the intestinal epithelium. The dysbiosis pattern induced by PFOS and PFOA resembles that observed in pathological conditions including colitis, obesity, and metabolic syndrome, supporting a functional link between PFAS exposure, microbiota disruption, and intestinal inflammation [12].

### 5.2. Microbiota–Host Metabolic Interactions and Associated Diseases

Beyond taxonomic shifts, PFAS perturb the intestinal metabolome, the dynamic pool of metabolites generated through host–microbe cross-talk, leading to functional consequences, frequently associated with disease development.

In PFAS-exposed murine models, a significant reduction in short-chain fatty acids (SCFA) has been reported [66]. These microbial metabolites are essential for epithelial integrity, functioning as energy substrates for colonocytes and modulating inflammatory signaling. A decline in SCFA production reduces the anti-inflammatory potential of the mucosa and interferes with GPR43/41-mediated signaling pathways, thereby compromising barrier integrity, leading to increased intestinal permeability [86]. In addition, PFAS exposure leads to elevated hepatic enzymes such as Alanine aminotransferase (ALT) and Aspartate aminotransferase (AST) and a decrease in their ratio (ALT/AST) in mice. Serum levels of these enzymes are markers of dyslipidemia; thus, their elevation suggests that PFOS may induce dyslipidemia in murine models [12], while a lower ALT/AST ratio has been associated with increased cardiovascular disease risk [87]. The exposure of human colonic models to a PFAS mixture increased the production of SCFAs, together with significant alterations in other metabolites, including a decrease in acetophenone and taurocholic acid in both treatments compared to controls [84]. These results suggest that PFAS exposure can interfere with gut microbiota homeostasis and affect the production of microbial metabolites relevant to metabolism and physiological balance.

PFAS can significantly influence lipid metabolism through their interaction with nuclear receptors of the PPAR family, which regulate the expression of genes involved in lipogenesis, fatty acid oxidation, and adipocyte differentiation [88]. PPAR activation by PFAS may therefore contribute to changes in hepatic and systemic lipid composition, including increased cholesterol levels and triglyceride accumulation, effects observed in both experimental studies and epidemiological investigations. Emerging evidence suggests that the gut microbiota may modulate these metabolic processes through the production of SCFAs, which act as regulatory cofactors of PPARs and may amplify PFAS-induced lipid alterations [88]. This interaction between PFAS exposure, nuclear receptor regulation, and microbial metabolism highlights an integrated mechanism through which PFAS can disrupt lipid homeostasis and contribute to systemic metabolic dysfunction.

PFOS and GenX exposure enriched metabolic pathways in the small intestine microbiota related to amino acid biosynthesis, including aromatic amino acids (AAA), branched-chain amino acids (BCAA), and pathways involving arginine and lysine [9]. Elevated AAA and BCAA levels have been linked to metabolic and cardiovascular disorders, such as Metabolic Liver disease and obesity [89], insulin resistance and type 2 diabetes [90], and heart failure [91]. Similarly, altered arginine and lysine metabolism can disrupt host homeostasis [92,93], while increased arginine metabolic activity has been associated with coronary heart disease risk [94]. For GenX, enhancement of glutamate and glutamine pathways, purine degradation, and cobalamin biosynthesis suggests potential links with neurodegenerative disorders such as Alzheimer’s disease, and with hyperuricemia and oxidative stress [95,96,97,98,99]. Systemically, both PFOS and GenX elevated cortisol and carnitine, known markers of oxidative stress [100], and decreased L-theanine, L-(−)-threonine, alanine-glutamate, and glycine-threonine levels, suggesting inhibition of amino acid biosynthetic pathways [101]. Exposure to PFOS was found to be associated with reduced levels of prostaglandin F2, suggesting potential interference with endocrine regulatory pathways [102]. These metabolic alterations indicate that PFAS impacts extend far beyond alterations in microbial composition, deeply affecting functional interactions between the microbiota and intestinal epithelial cells.

Epidemiological studies have identified associations between high serum PFAS levels and increased incidence of chronic IBD [8]. Although causality remains to be fully established, current evidence suggests that PFAS-induced dysbiosis, combined with epithelial barrier impairment and enhanced production of pro-inflammatory metabolites, constitutes a plausible pathogenic mechanism. In particular, the reduction in beneficial genera such as Akkermansia muciniphila may impair mucus turnover and mucosal regeneration, whereas the expansion of Proteobacteria enhances systemic LPS release [103,104,105]. This pro-inflammatory microbial configuration may partly explain the elevated risk of chronic intestinal and metabolic disorders linked to PFAS exposure.

PFAS-induced microbiota perturbations are not restricted to the gastrointestinal environment. The combination of reduced SCFA synthesis and increased systemic LPS translocation promotes chronic endotoxemia, characterized by TLR4 activation and pro-inflammatory cytokine production in peripheral organs, including liver and brain [12,66]. This condition contributes to metabolic and neuro-inflammatory dysfunctions, defining a gut–liver–brain axis highly vulnerable to PFAS exposure.

### 5.3. Evidence from Alternative Model Organisms

PFAS-induced microbial alterations are not limited to mammalian systems. Experiments in Zebrafish exposed to PFAS for 21 days revealed marked shifts in gut microbial composition and diversity, reducing the abundance of opportunistic genera such as Aeromonas, Plesiomonas, and Cetobacterium, while increasing the prevalence of Shewanella and Vibrio [106]. These microbial shifts are associated with elevated markers of intestinal inflammation and oxidative stress, indicating an evolutionarily conserved toxic response to PFAS. Another relevant in vivo alternative model used to investigate PFAS-mediated gut toxicity is the silkworm [107]. PFAS exposure alters their gut microbiota diversity, increasing the relative abundance of Proteobacteria and Actinobacteriota while decreasing Firmicutes, a profile consistent with dysbiosis. Furthermore, this study confirmed that PFAS-induced dysbiosis is accompanied by significant metabolic dysfunction within the gut microbial community of the silkworm. These alternative organisms confirm that PFAS-induced microbial disruptions are consistent across species, underscoring their value for mechanistic research and supporting the value of non-mammalian systems for mechanistic investigations of microbiota-dependent environmental toxicity.

## 6. Clinical and Epidemiological Evidence

**Figure 1:**
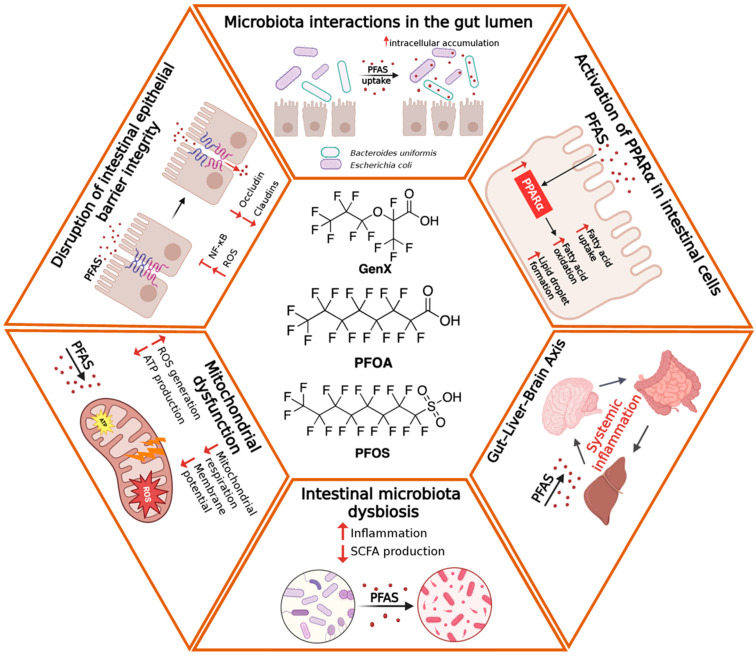
Schematic overview of the main intestinal alterations associated with PFAS exposure. Long- and short-chain PFAS reach the intestinal lumen primarily through oral intake and directly interact with the epithelial barrier. Exposure induces tight junction disruption, increased intestinal permeability, oxidative stress, mitochondrial dysfunction, and altered epithelial differentiation. Concurrently, PFAS promote metabolic impairments (as for instance lipid dysregulations) and gut dysbiosis, characterized by the expansion of pro-inflammatory bacteria and the reduction in beneficial taxa, ultimately contributing to local inflammation and bi-directional (Intestine-Brain) systemic effects.

Although experimental systems have long represented the main source of information on PFAS-induced intestinal toxicity, recent clinical and epidemiological studies are increasingly shedding light on their relevance to human health. According to the previous section, the main intestinal alterations associated with PFAS exposure have been schematically depicted in Figure 1.

Most human data derive from epidemiological investigations in communities exposed to PFAS-contaminated drinking water or food. Such studies consistently report associations between elevated serum PFAS concentrations and chronic gastrointestinal disorders, notably IBD [8,108]. Nevertheless, evidence of a direct cause-and-effect relationship remains limited, primarily due to the difficulty of quantifying cumulative exposure and controlling interactions with other environmental factors. However, dedicated clinical investigations addressing PFAS effects on intestinal barrier function, particularly those employing molecular biomarkers or advanced imaging techniques, remain limited. In this context, identifying sensitive biomarkers of PFAS-induced intestinal dysfunction and developing controlled longitudinal cohorts is crucial to bridge the gap between experimental data and epidemiological observations and provide a solid basis for a more accurate assessment of human risk.

In a recent cohort study, it has been observed that maternal PFAS concentrations during pregnancy correlated with variations in maternal weight during the perinatal period [109], which is a marker of long-term cardiometabolic health [110]. Specifically, higher serum levels of PFOA were associated with less weight loss in the first year after delivery and more significant weight gain over the subsequent three years. This relationship appears to be more evident among women with a higher pre-pregnancy Body Mass Index, suggesting that this group is more susceptible to the potential long-term metabolic effects of PFAS exposure. Excessive or insufficient weight gain during pregnancy has been linked to an increased risk of children developing obesity, hypertension, and insulin resistance during childhood [111,112].

The association between exposure to PFAS and the downregulation of specific microRNAs (miRNAs) in a selected population has been investigated in the Ronneby cohort of Sweden [113]. This municipality has experienced very high levels of PFAS exposure through drinking water since the mid-1980s. They observed that PFAS may exert some of their toxic effects by altering miRNA expression profiles and influencing the post-transcriptional control of numerous regulatory genes. In silico functional analyses also indicate a possible interconnection between serum PFAS levels, miRNA modifications and the regulation of biological pathways involved in chronic diseases, including cardiovascular disease, neurodegeneration (e.g., Alzheimer’s disease) and tumor proliferation.

The strongest epidemiological evidence relates to the link between exposure to PFAS and the onset of chronic IBD. Epidemiological analyses indicate that individuals with higher serum concentrations of PFOS and PFOA are at an increased risk of developing IBD, particularly Crohn’s disease, compared to unexposed populations [8]. The chronic nature of exposure and the long biological half-life of PFAS make a temporal correlation between their accumulation and persistent intestinal inflammation plausible. Mechanistic interpretations point toward PFAS-induced dysbiosis and altered mucosal immune signaling, processes corroborated by multiple experimental animal models [9,106,107,114]. Notably, a PFAS-induced increase in Proteobacteria and a decrease in SCFA-producing bacteria likely promote a chronic pro-inflammatory environment [66,70]. Following exposure to a mixture of PFAS in humans, downregulation of genes associated with tight junctions has also been observed [5,6]. This indicates a compromised intestinal barrier and significant alterations in the composition of the gut microbiota and the biosynthesis of steroid hormones. While the causal relationship has yet to be definitively proven, the correlation between human observations and preclinical data strengthens the hypothesis of a biologically plausible link.

In addition to their direct involvement in the gastrointestinal tract, PFAS appear to influence systemic pathological conditions of multifaceted nature via mechanisms involving the intestinal barrier. For instance, longitudinal clinical studies have reported an increased incidence of metabolic syndrome, dyslipidemia and type 2 diabetes with chronic exposure to PFAS [68,115]. Alteration of the intestinal barrier resulting in subclinical endotoxemia could be a mechanism linking environmental exposure to metabolic disorders. Indeed, the translocation of LPS into systemic circulation due to intestinal barrier alteration stimulates hepatic production of pro-inflammatory cytokines and alters insulin signaling [75,116]. Furthermore, the reduced availability of SCFAs, essential for regulating lipid and carbohydrate metabolism, observed in mouse models exposed to PFAS [66] contributes to chronic metabolic inflammation [117].

Despite the growing body of evidence, there are significant methodological limitations in clinical and epidemiological research on PFAS. Most of the currently available studies are cross-sectional and are based on correlations between serum levels and clinical parameters without precisely defining cumulative exposure. Furthermore, given their long half-life, blood levels of PFAS mainly reflect past exposures, complicating the assessment of temporal cause and effect. Additionally, co-exposure to other persistent pollutants, such as microplastics, pesticides, or heavy metals, may confound associations, complicating the isolation of PFAS-specific effects in epidemiological datasets. To overcome these limitations, longitudinal cohort studies that integrate environmental and biological biomonitoring with direct assessments of intestinal function (including multi-omic analyses) should be promoted.

### Developing Strategies for the Treatment of PFAS-Induced Intestinal Damage

As extensively discussed, it is evident that exposure to PFAS contributes to intestinal damage through alteration of the epithelial barrier, disruption of the gut microbiota, and activation of inflammatory pathways. Although therapies specifically approved for PFAS-induced intestinal toxicity are still lacking, several potential strategies are currently under investigation. The pharmacological approaches currently under consideration are mainly aimed at controlling the immune-inflammatory consequences induced by PFAS exposure. In this context, biologics and small molecules commonly used in IBD represent a rational option to limit chronic inflammation in exposed individuals. Among them, monoclonal antibodies targeting IL-23, such as mirikizumab, received the USA Federal and Drug Administration (FDA) approval in 2023 since it can reduce T-helper 17 cell activation through inhibition of IL-23 [118]. Other therapies commonly used in the clinical management of IBD include anti-TNF drugs. Of interest, in preclinical settings, mice treatment with anti-TNF drugs was able to restore the integrity of the intestinal barrier compromised by PFOA, including reinforcement of tight junctions, replenishment of the mucus layer, and promotion of intestinal stem cell function [119]. Moreover, this approach resulted in suppression of the NLRP3 inflammasome and PFOA-induced apoptosis. Even one month after treatment, the main inflammatory cytokines remained significantly elevated in PFOA-exposed mice but were markedly reduced following anti-TNF treatment. Increasing interest has also focused on compounds that strengthen the intestinal barrier by modulating the expression of tight junction proteins. For instance, itaconic acid, a small molecule metabolite and renewable organic acid, alleviated PFOA-induced intestinal damage, in part by restoring the expression of tight junction proteins and consequently reducing intestinal permeability in laying hens [120]. Itaconic acid can activate the Keap1/Nrf2 pathway, thereby regulating the downstream expression of antioxidant and anti-inflammatory genes [121]. Supplementation with itaconic acid in laying hens increased the activity of antioxidant enzymes such as glutathione peroxidase, superoxide dismutase, and catalase, and improved the mucosal barrier damaged by PFOA by restoring goblet cell counts and increasing the expression of MUC-2, a mucin family protein that is a fundamental component of the intestinal mucus layer [120]. In this respect, the use of antioxidants to counteract oxidative stress and mitochondrial damage associated with PFAS exposure may be considered an additional strategy to be integrated with other therapies. Compounds such as polyphenols, flavonoids, N-acetylcysteine, and other scavengers of reactive oxygen species may attenuate intestinal inflammation and improve barrier function [122,123].

In recent years, PFAS-induced dysbiosis has made the gut microbiota a key therapeutic target. Indeed, dietary patterns, probiotics-including butyrate-producing strains-or defined bacterial consortia, are being investigated for their ability to restore microbial balance, improve epithelial integrity, and modulate immune responses. Under conditions of PFAS exposure, these approaches may thus serve as effective adjuvant interventions. For instance, within the probiotic family, Lactobacillus rhamnosus has been extensively studied in the past due to its ability to tolerate the digestive environment, colonize, and survive in the animal intestine [124]. When administered to Zebrafish exposed to PFOA and PFOS (0.3 and 30 mg/L), this probiotic improved dysbiosis, with an increase in the abundance of Firmicutes and Actinobacteria and a decrease in Proteobacteria within the intestine [125]. In addition, it restored intestinal villus numbers, thereby improving intestinal barrier integrity. Prebiotics, fermentable fibers and selective oligosaccharides can also have beneficial effects by promoting the growth of bacteria and the production of anti-inflammatory metabolites. Inulin, a fructan with linear chains of fructosyl groups connected by β(2-1) glycosidic bonds, exerted a strong modulatory effect on the gut microbiota by stimulating the growth of beneficial bacteria such as Bifidobacterium and Lactobacillus [126]. Inulin was recently shown to mitigate intestinal toxic effects induced by PFOA and GenX in both dams and mouse offspring [3]. Specifically, this prebiotic significantly modulated the gut microbiota by increasing the relative abundance of beneficial bacteria, such as Lieibacterium and Bifidobacteriaceae. In addition, the relative abundance of potentially harmful bacteria, such as Turicibacter, was significantly reduced, further highlighting the positive observed effects.

In summary, potential strategies for PFAS-induced intestinal damage lie at the intersection of immunomodulation, microbiota restoration, and protection against oxidative stress. Although many of these approaches are still in the experimental phase, their development represents a promising direction for future personalized interventions in individuals exposed to PFAS.

## 7. Conclusions

**Figure 2:**
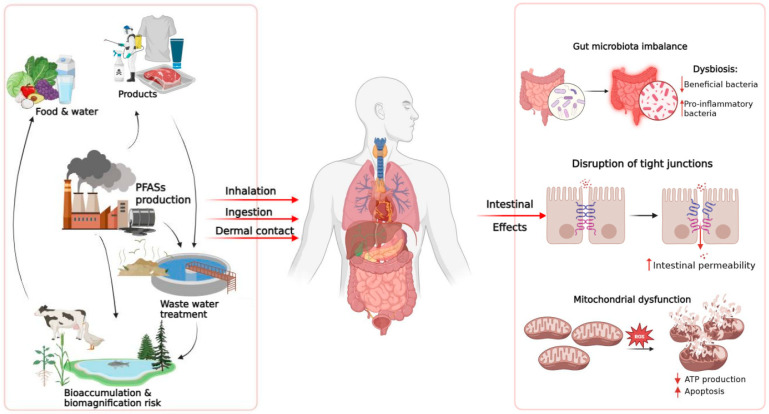
Summary of the main routes of exposure to PFAS and their effects on the intestines. PFAS from environmental sources, food, and consumer products enter the human body through inhalation, ingestion, or skin contact. Once absorbed, PFAS can alter intestinal homeostasis through various mechanisms, including dysbiosis of the intestinal microbiota, increased epithelial permeability due to compromised tight junctions, and mitochondrial dysfunction characterized by increased ROS, reduced ATP production and increased cell apoptosis.

Growing experimental and epidemiological evidence identifies the intestine as a central target of PFAS toxicity. Their amphiphilic nature, remarkable chemical stability, and high binding affinity for plasma proteins enable PFAS to penetrate biological membranes and accumulate within intestinal tissues. Collectively, the available data portray PFAS as multifactorial disruptors of intestinal homeostasis, acting through convergent structural, immune, and metabolic mechanisms. Figure 2 and Table 1 provide a concise overview of the main exposure pathway and intestinal and systemic effects of PFAS, combining mechanistic insights from experimental studies with emerging human evidence. In summary, following absorption, PFAS disrupt epithelial homeostasis by suppressing the expression of crucial tight-junction proteins (occludin, claudins, and Tjp1) and by triggering oxidative stress–mediated apoptotic pathways. These structural alterations activate inflammatory cascades driven by NF-κB and inflammasome pathways (NLRP3, AIM2), sustaining cytokine secretion and immune-cell infiltration. Beyond epithelial injury, PFAS exposure reprograms intestinal metabolism through PPAR-α activation and mitochondrial dysfunction, disrupting lipid oxidation, cellular energetics, and regenerative capacity. Metabolic perturbations frequently parallel microbiota dysbiosis, characterized by a reduced Firmicutes/Bacteroidetes ratio and loss of beneficial taxa such as Lactobacillus and Akkermansia, amplifying mucosal inflammation and systemic metabolic imbalance [3,9,66]. Noteworthy, gut microbiota dysbiosis and intestinal barrier dysfunction are linked through a bidirectional, self-reinforcing interaction. Qualitative and functional alterations of the gut microbiota, particularly the reduction in SCFA-producing bacteria, lead to diminished metabolic support to the intestinal epithelium and to impaired expression and organization of tight junction proteins, resulting in increased intestinal permeability [127,128]. Loss of barrier integrity facilitates the translocation of microbial components and the activation of local inflammatory responses, which in turn further remodel microbiota composition and reduce its functional stability [129]

Given this picture, the intestine should be regarded not merely as an absorption interface but as an active hub connecting environmental PFAS exposure to systemic disease processes. Also, investigations should combine longitudinal biomonitoring with mechanistic and multi-omics approaches to establish causal relationships and refine exposure thresholds, particularly for short-chain and emerging PFAS. The complexity of interactions between PFAS, the intestinal epithelium, and the microbiota requires an integrated and multidisciplinary research approach that combines cell-based systems, animal models, and human studies. The coordinated use of these experimental platforms enables a more precise characterization of PFAS effects on the intestinal barrier by linking structural alterations with functional and metabolic changes. Such a comparative approach also increases translational relevance, providing a more reliable assessment of the potential health risks for exposed human populations.

### Future Directions

Despite substantial advances, major knowledge gaps remain regarding the intestinal toxicity of PFAS, especially short-chain derivatives and next-generation analogues. Future studies should aim to:

- Clarify mechanistic pathways: determine how variations in fluorinated chain length and terminal functional groups influence epithelial absorption, immune activation, and metabolic reprogramming, using well-established integrative in vitro and in vivo systems as well as novel intestinal organoids and gut-on-chip platforms.
- Assess realistic exposure scenarios: examine chronic, low-dose, and mixture exposures representative of environmental and dietary conditions encountered in human populations.
- Define microbiota-mediated mechanisms: apply integrative multi-omics (metagenomics, metabolomics, transcriptomics) to dissect how PFAS-induced dysbiosis drives intestinal inflammation and barrier dysfunction.
- Strengthen translational research: develop prospective human cohorts correlating PFAS exposure biomarkers with intestinal permeability, immune, and metabolic parameters, while standardizing analytical workflows for PFAS quantification in biological samples.
- Explore mitigation strategies: investigate nutritional, probiotic, and pharmacological interventions aimed at restoring microbial diversity and reinforcing epithelial barrier integrity under chronic PFAS exposure.

In conclusion, integrating mechanistic, functional, and clinical dimensions will be critical to comprehensively elucidate PFAS intestinal toxicity and to guide future regulatory and public health interventions.

## Figures and Tables

**Table 1:** Summary of key mechanisms and health implications of PFAS toxicity.

Aspect	Main Findings	Implications
Chemical nature and persistence	PFAS are highly stable, amphiphilic compounds with strong C–F bonds, resistant to degradation and prone to bioaccumulation.	Long-term persistence in environment and tissues enables chronic human exposure.
Exposure routes	Predominantly oral, via contaminated water, food, and indoor dust; PFAS detected in serum, urine, and breast milk.	Continuous low-dose intake contributes to cumulative systemic burden.
Epithelial damage	Downregulation of tight-junction molecules (occludin, claudins, Tjp-1); oxidative stress and apoptosis; altered stem-cell renewal.	Increased intestinal permeability (“leaky gut”), chronic inflammation, impaired regeneration.
Inflammatory and immune response	Activation of NF-κB, NLRP3, AIM2; TNF-α and IL-6 release; macrophage and neutrophil infiltration.	Sustained mucosal inflammation; potential contribution to IBD and metabolic inflammation.
Metabolic alterations	PPAR-α–dependent lipid reprogramming and mitochondrial dysfunction reduce ATP synthesis and energy balance.	Links PFAS exposure to dyslipidemia, obesity, and insulin resistance.
Microbiota dysbiosis	Reduced Firmicutes/Bacteroidetes ratio; depletion of Lactobacillus and Akkermansia; loss of SCFA producers.	Amplifies inflammation and endotoxemia; may drive gut–liver–brain metabolic axis dysfunction.
Experimental evidence	Data from in vitro (Caco-2, organoids) and in vivo models (mice, Zebrafish, Drosophila, silkworms) reveal consistent gut toxicity.	Mechanisms are evolutionarily conserved, supporting translational validity.
Human evidence	Associations between serum PFAS levels and IBD, metabolic syndrome, and perinatal outcomes.	Suggests biologically plausible links between chronic exposure and systemic disease.
Research priorities	Need for multi-omics, low-dose mixture studies, longitudinal human cohorts, and mitigation strategies.	Essential for defining causal mechanisms and exposure thresholds.

## Data Availability

No new data were created or analyzed in this study. Data sharing is not applicable to this article.

## Abbreviations

The following abbreviations are used in this manuscript:

AAA	Aromatic amino acids
AIM2	Absent in melanoma 2
ALT	Alanine aminotransferase
AST	Aspartate aminotransferase
BCAA	Branched-chain amino acids
BMD	Benchmark dose
EFSA	European Food Safety Authority
EPA	Environmental Protection Agency
FDA	Food and Drug Administration
GenX	Perfluorohexanoic acid
IBD	Inflammatory bowel diseases
IL	Interleukin
LPS	Lipopolysaccharide
miRNAs	microRNAs
NF-κB	Nuclear factor kappa-light-chain-enhancer of activated B cells
NLRP	NOD-, LRR- and pyrin domain-containing protein
NOAEL	No Observed Adverse Effect Level
PFBA	Perfluorobutanoic acid
PFAS	Per- and Polyfluoroalkyl substances
PFBS	Perfluorobutane sulfonic acid
PFOA	Perfluorooctanoic acid
PFOS	Perfluorooctane sulfonate
PPARα	Peroxisome proliferator-activated receptor α
PKC	Protein kinase C
ROS	Reactive oxygen species
SCFAs	Short-chain fatty acids
TLR	Toll-like receptor
TNF	Tumor necrosis factor
Tjp	Tight junction protein
